# Acetic Acid Production from *Aspergillus terreus* Isolated from Some Agricultural Soils Collected from Selected Locations within the North Gondar Zone, Amhara Region, Ethiopia

**DOI:** 10.1155/2024/5183336

**Published:** 2024-08-05

**Authors:** Kidist Alemayehu, Tamene Milkessa Jiru, Nega Berhane

**Affiliations:** ^1^ Department of Biology College of Natural and Computational Sciences University of Gondar, P.O. Box: 196, Gondar, Ethiopia; ^2^ Department of Environmental and Industrial Biotechnology Institute of Biotechnology University of Gondar, P.O. Box: 196, Gondar, Ethiopia; ^3^ Department of Medical Biotechnology Institute of Biotechnology University of Gondar, P.O. Box: 196, Gondar, Ethiopia

## Abstract

Acetic acid, a substance with numerous uses as a bulk chemical, may be produced novelty by *Aspergillus terreus*. With the newfound understanding of *Aspergillus* species catabolism of glucose, fermentation techniques for the generation of secondary metabolites like acetic acid in the Ethiopian north Gondar zone can be developed with glucose feeding and pH feedback management. Previous works done on extracting organic acids including acetic acid from filamentous fungi in Ethiopia and at the global level are scanty. Therefore, this study aimed to produce acetic acid from *A. terreus* isolated from agricultural soils in the north Gondar zone of Ethiopia using barely straw as a substrate. In the current study, *Aspergillus* isolates were obtained in the samples taken from three different locations. The isolates were screened for acetic acid production. The optimum temperature and pH for the maximum production of acetic acid by the selected isolate were also undertaken. The potential isolates were further cultured using barley straw as a local substrate. Preliminary identification of the selected isolates was based on morphological methods. Molecular characterization (amplification and sequencing of the two intergenic spacers, ITS1 and ITS2, and the intervening 5.8S gene of the ribosomal RNA) was carried out to confirm the identity of the *Aspergillus* isolates. When the isolates were screened for the production of acetic acid, an isolate from low land (isolate LL_2_) had the highest yield (72.5 ± 1.65 g/l) on basal screening media. The optimum temperature and pH for the maximum production of acetic acid by this isolate were 30°C and pH 5.0. A sequence similarity of 98.5% to *A. terreus* isolate LL_2_ (KIA) was obtained by comparing the *Aspergillus* isolate to a reference sequence in the GenBank using the BLAST algorithm. It can be concluded from this study that *A. terreus* isolated from agricultural soil in the north Gondar zone of Ethiopia could produce more acetic acid using barely straw as a substrate.

## 1. Introduction

For their potential industrial uses and their function in natural ecology, the synthesis of organic acids from filamentous fungi has gained much attention [1]. Organic acids are the third-largest category among organic compounds. Organic acids are the most adaptable components in the food and beverage sectors. Some of the common organic acids utilized in numerous industries include fumaric acid, acetic acid, lactic acid, formic acid, tartaric acid, malic acid, and gluconic acid. There may be a wide range of industrial uses for organic acids. They can be used as chemical reagents, fungicides, herbicides, microbicides, pH adjusters, ingredients in the synthesis of drugs, and food additives. In the culinary, agricultural, cleaning, and cosmetics industries, organic acids are employed as solvents.

Acetic acid (CH_3_COOH), also known as ethanoic acid, is the most significant carboxylic acid [2]. Vinegar is a salt, ester, or acylal of acetic acid; acetate is a dilute (about 5% by volume) solution of acetic acid formed by fermentation and oxidation of natural carbohydrates. In the manufacturing industry, acetic acid is used to create metal acetates, which are sometimes used in printing processes. Vinyl acetate is a colorless organic compound used to make plastics. On the other hand, cellulose acetate is used to make photographic films and textiles, and volatile organic esters, which are frequently used as solvents for resins, paints, and lacquers. Acetic acid is a crucial metabolic step in biology that naturally occurs in plant juices and body fluids [3]. Vinegar, which is made from acetic acid produced by double fermentation, is mostly used in the food business. Vinegar is a sour-tasting liquid. These days, there are more inventive ways to adapt vinegar use to the modern way of life and food culture. A variety of acetic acid concentrations are employed as food preservatives against food spoilage and to enhance the flavor of foods with a longer shelf life. Antimicrobial coatings that are both edible and inedible have also become popular new uses [4]. Global demand for various organic acids is rising. Similar to this, Ethiopia has a high demand for organic acids, particularly acetic acid, which is used in a wide range of downstream sectors, including textiles, food processing, pharmaceuticals, and quite a few other industrial chemical processes. However, organic acids are imported from abroad. For instance, the nation buys acid from several countries, including India, Saudi Arabia, China, Germany, Kenya, and the United States. For purchasing and transporting these organic acids, a significant amount of foreign currency is spent. It has become increasingly common for the manufacturing industry to rely on imported organic materials. These typically have an effect on the country's GDP, trade rate, and inflation rate.

Additionally, due to lack of foreign cash, various enterprises and industrial sectors that require this acetic acid are unable to get it. Numerous microbes have been employed over time to produce acetic acid. The most commonly utilized microorganisms for the production of acetic acid are acetic acid bacteria in the genera of *Acetobacter* and *Gluconobacter* [5]. Although several species of microorganisms, including filamentous fungi, produce acetic acid in significant amounts (50.0–70.0 g/l), they have not been exploited for industrial production up until recently. A filamentous fungus, *A. terreus,* is still the desired microorganism in industrial manufacture. Due to their ease of handling, low-cost downstream processing, and high and consistent yields, *A. terreus* strains are useful acetic acid and other organic acid producers and help keep the processes cost-competitive. Taking the above issues into consideration, this study aimed to produce acetic acid from *A. terreus* isolated from agricultural soils in the north Gondar zone of Ethiopia using barely straw as a substrate.

## 2. Materials and Methods

### 2.1. Sample Collection

**Figure 1:**
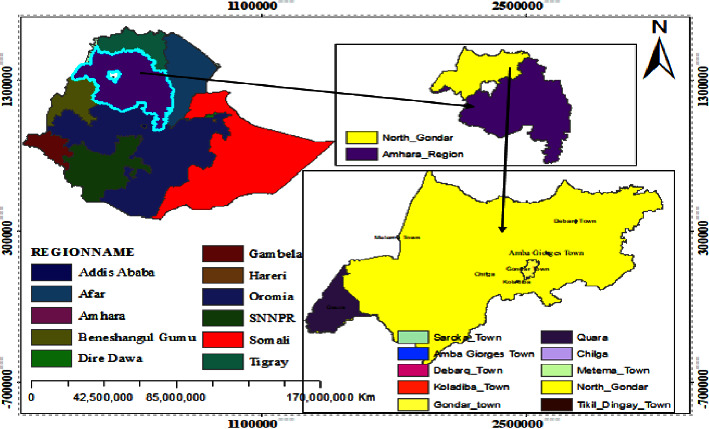
Study area location: north Gondar zone, Amhara regional state, Ethiopia (GIS map by Kidist Alemayehu).

In the current study, soil samples were collected from different agricultural fields in high-land (HL), midland (MD), and low-land (LL) locations of the north Gondar zone, Amhara regional state, Ethiopia (Table 1 and Figure 1). Sterile plastic bags were used to collect soil samples, which were then brought to the laboratory of the Department of Biology, College of Natural and Computational Sciences, University of Gondar. To prevent microbial dynamics, plastic bags containing soil samples were kept in an ice box while transporting the samples.

### 2.2. Isolation of Filamentous Fungi

About 1 g of each soil sample was suspended in 9 ml of sterile saline (0.9% w/v) before being processed. Serial dilutions were done from 10-1-10-5. Then, 0.1 ml mixture from the last dilution was spread onto the surface of solid potato dextrose agar (PDA) media using a bent glass rod and incubated at 30°C temperature. Everyday observations were conducted, while the plates were incubated at 30°C for 7 days to check the presence of filamentous fungi. By repeatedly subculturing on PDA supplemented with chloramphenicol, pure cultures of isolates were obtained by growing them at 30°C. Daily observations of the cultures were made, and fungal growth was subcultured on new PDA plates until pure isolates were obtained. Pure cultures were preserved on PDA slants at 4°C for further use and subcultured every four weeks to sustain them [6].

### 2.3. Morphological Identification of Filamentous Fungi

The isolated fungi were identified following the procedure of Domsch et al. [7]. Their macroscopic (color, texture, and appearance) and microscopic features were evaluated after the development on PDA media. An inoculating needle that was burned over a flame of Bunsen burner was used to transfer the outgrowth from the culture plate to a drop of cotton blue lactophenol on the slide. To prevent crushing and crowding of the mycelium, samples were carefully collected using an inoculating wire loop. For microscopic identification, samples were viewed under ×40 compound binocular microscope objective. The characterized fungal isolates were then used in the subsequent investigations.

### 2.4. Fungal Strain and Inoculum Preparation

The fungal strains were kept in spore form in a 70% (v/v) glycerol stock at −20°C temperature (deep freeze). Czapek-Dox agar (Sigma) plates were used to maintain the stock culture. Conidiospores were obtained by shaving and extracting spores with sterile water containing 0.04% (v/v) Tween 80 from seven-day culture on Czapek-Dox agar plates that had been incubated at 30°C. The spore suspension was adjusted to the desired concentration by diluting it with sterile deionized water, which was 10^6^ spores/ml of the medium. Adjustment (quantification) of conidia was carried out with the help of a spectrophotometer at 560 nm.

### 2.5. Screening Fungal Isolates for the Production of Acetic Acid

The generation of acetic acid was detected using a Czapek-Dox broth medium as an acid indicator, in which the fungi were cultured for seven days to detect the development of a yellow color in a medium, a sign that acid was being produced. In addition, the isolates were screened quantitatively for the production of acetic acid by inoculating 1 ml of 5-day-old spore suspension in sterile liquid basal medium containing (g/l): sucrose (30), sodium nitrate (3), magnesium sulfate (0.5), potassium chloride (0.5), potassium phosphate dibasic (1), and ferrous sulfate (0.01) with pH adjusted to 5. Fermentation was carried out in 250 ml Erlenmeyer flasks containing 100 ml fermentation medium at room temperature. The concentration of acetic acid in the fermentation medium was estimated titrimetrically [8, 9] after 48 hours of incubation.

### 2.6. Substrate and Basal Medium Preparation

The substrate, namely, barely straw, was treated following the procedure of Egbe et al. [10]. Grounded barley straw was pretreated using 5 N HCl acid as a slurry at a solid-to-liquid ratio of 1 : 10 (w/v) and incubated in water bath at 106°C for 1 hour. After cooling, the substrate was washed with distilled water and dried in an oven at 106°C. On the other hand, the basal medium formulated by Tangnu et al. [11] was prepared by dissolving (NH_4_)_2_SO_4_ (1.4 g), KH_2_PO_4_ (2 g), MgSO_4_·7H_2_O (0.3 g), CaC_l0_·2H_2_O (0.4 g), NH_2_CONH_2_ (0.3 g), proteose peptone (1 g), Tween 80 (0.2 ml), FeSO_4_·7H_2_O (5 mg), MnSO_4_·H_2_O (1.6 mg), ZnSO_4_·7H_2_O (1.4 mg), and CoCl_2_ (2 mg) in a liter of deionized water.

### 2.7. Optimization of Fermentation Medium

Optimization of cultivation conditions for the production of acetic acid by the screened filamentous fungus was undertaken. The optimized parameters include temperature, ethanol concentration, and pH. After the inoculated medium was incubated for one day, the amount of acetic acid produced was determined. For the generation of acetic acid by the filamentous fungus, different temperatures were chosen, including 25, 30, 35, 40, and 45°C, as well as a range of ethanol concentration starting at 5, 6, 7, 8, 9, 10, 11, and 12 [10]. On the other hand, the effect of pH on the production of acetic acid by the filamentous fungus was evaluated at different pH values, including 3, 3.5, 4, 4.5, 5, 5.5, 6, 6.5, and 7. The titrimetric method was used to determine the amount of acetic acid present in the fermentation medium [12].

### 2.8. Fermentation

Acetic acid was produced in accordance with Shafi et al. [13]. The basal medium was mixed with 10 g of treated crude barley straw and moistened with the appropriate amount of distilled water. The moisture content of the basal medium was adjusted to 65% (w/v). Fermentation experiments were carried out in 250 ml flasks. After autoclaving at 121°C for 15 minutes, the substrate's pH initially adjusted to 5 with 2 N NaOH and 2 N HCl. The flasks were cooled to room temperature and injected with 1 ml (10^6^ spores/ml) of spore suspension. The culture was incubated for five days at 30°C. Following the filtration process, acetic acid concentration in the medium was measured titrimetrically using the filtrate [14]. The concentration of acetic acid was determined using the titration procedure described by Sharafi et al. [13].

#### 2.8.1. Molecular Characterization

To confirm the identity of the morphologically characterized fungal isolate, molecular analysis was done. For such molecular analysis, DNA extraction,DNA extraction, PCR amplification, gel electrophoresis, sequencing, and phylogenetic tree analysis of the two intergenic spacers, ITS1 and ITS2, and the intervening 5.8S gene of the ribosomal RNA were done.

#### 2.8.2. DNA Extraction

The filamentous fungus that was screened and selected for the production of acetic acid was first grown on the PDA medium at 30°C for 72 hours. After incubation, the fungal mycelial mass from the culture plate was taken and ground in a sterile mortar and pestle using 500 *µ*l extraction buffer [100 mM Tris-HCl (pH 8.0), 20 mM EDTA (pH 8.0), 1.4 M NaCl, 2% CTAB, and 0.2% 2-Mercaptoethanol], centrifuged at 14,000 rpm for 10 minutes. Genomic DNA was extracted from the isolated filamentous fungus pellets using GenElute™ Plant Genomic DNA Purification Kit (Sigma-Aldrich) according to the manufacturer's instructions. The DNA was stored at −20℃ and used as a template for polymerase chain reaction (PCR) amplification.

#### 2.8.3. Polymerase Chain Reaction (PCR) Amplification

The ITS rDNA and 5.8S rDNA regions of fungal isolate were amplified using a Perkin Elmer 2400 thermal cycler following the protocol of Gene et al. [14]. White et al. [15] reported the primer pairs used: ITS1 forward primer (5′-TCCGTAGGTGAACCTGCGG-3′) and ITS4 reverse primer (5′-TCCTCCGCTTATTGATATGC-3′). The PCR mix (25 *µ*l of PCR master mix solution) with 4 *μ*l template DNA and 1 *μ*l of each primer was prepared and performed according to the recommendations of Promega, United States. The amplification steps consisted of predenaturation at 94°C for 5 minutes followed by 35 cycles consisting of denaturation at 94°C for 50 seconds, annealing at 52°C for 1 minute, extension at 72°C for 40 seconds, and a final extension of 5 minutes at 72°C [16].

#### 2.8.4. Gel Electrophoresis

A 1.5% agarose gel was used to run the amplified DNA. With the aid of a UV transilluminator (gel documentation system), DNA bands were seen and photographed. A 100-bp DNA ladder (Gibco-BRL) was placed into the first lane of the agarose gel, and the amplicon size was compared with the DNA ladder to determine whether the target gene (ITS1-5.8S-ITS2 rRNA gene) had been amplified.

#### 2.8.5. Sequencing and Phylogenetic Tree Analysis

PCR-amplified products of filamentous fungal isolate were sent to Macrogen Company, Netherlands, and were sequenced in both forward and reverse directions using ITS1 primer (TCCGTAGGTGAACCTGCGG) and ITS4 primer (TCCTCCGCTTATTG ATATGC), respectively, using the Sanger sequencing method. After receiving sequenced PCR-amplified products of fungal isolates, their sequence results of forward and reverse primers were edited using BioEdit software and their consensus region was obtained. Sequences were compared with reference sequences showing sequence homology in GenBank of the National Center for Biotechnology Information (NCBI) (https://www.ncbi.nlm.nih.gov) database using MEGA software, version 11.0. Homologous sequences in the GenBank were retrieved using the Basic Local Alignment Search Tool (BLAST) algorithm [17]. For phylogenetic tree analysis, the available gene sequence data of related organisms were retrieved in FASTA format and aligned using Clustal W [18]. The phylogenetic tree was constructed using the neighbor-joining (NJ) technique, and distances were computed with the help of the maximum composite likelihood method. The branching patterns were checked using 1000-bootstrap replicates. Finally, the sequencing finding was deposited in the NCBI database, and the accession number was obtained for LL_2_ (KIA). The accession number for the isolate is AcOR038984.

### 2.9. Statistical Analysis

All experiments were done in triplicates. The experimental results were expressed as mean ± standard deviation of the three replicates.

## 3. Results

### 3.1. Isolation and Screening of Acetic Acid Producing Filamentous Fungi

In the current study, a total of 50 soil samples were collected from different locations of north Gondar zone, Amhara regional state, Ethiopia. From these samples, about 15 colonies were obtained on PDA agar plates. Fungi that were isolated from the high-land, low-land, and midland parts of the study area were designated as HL (HL_1_–HL_7_), LL (LL_1_–LL_4_), and ML (ML_1_–ML_4_), respectively.

Then, the generation of acetic acid was detected using Czapek-Dox broth medium as acid indicator, in which the isolated filamentous fungi were cultured for seven days to detect the development of a yellow color in the medium, a sign that acid was being produced and the others were white in color in the medium in which acid was not produced. In addition, the amount of citric acid produced by the different filamentous fungal isolates was recorded after 48 hours of incubation. From the 15 isolates, three filamentous fungal isolates (HL_4_, LL_2_, and ML_1_) were found to produce maximum mean value of acetic acid after 48 hours of incubation and were selected for further studies. The amount of acetic acid produced by HL_4_, LL_2_, and ML_1_ was 29 g/l, 57 g/l, and 47 g/l, respectively.

### 3.2. Morphological Identification of Acetic Acid Producing Filamentous Fungi

**Figure 2:**
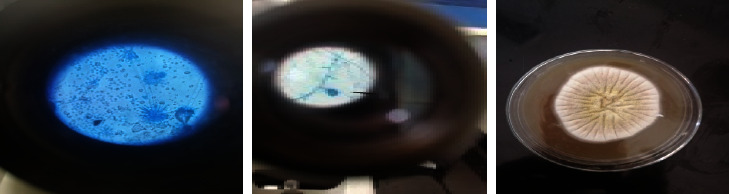
Matured *Aspergillus* as viewed under the microscope and macroscope (x40).

When isolates were first grown on PDA, their mycelia were white, but, as they grew and matured, they developed velvety thick yellow soluble pigment-colored spores. As indicated in Figure 2, the fungal morphology under this investigation on a macroscale is from buff to cinnamon (brown)-colored colonies. Yellow soluble pigments that can occasionally have a yellow reverse and tiny, smooth-walled, globose-shaped structure were recorded under a microscope. The formation of aleurioconidia, asexual spores larger than phial conidia (6–7 mm in diameter) and generated directly on the hyphae, is specific to this species. These features are found to be the characteristics of *A. terreus.*

### 3.3. Acetic Acid Production

In this study, acetic acid production capacity of filamentous fungal isolate was assessed using alternative raw material (barley straw) substrate under solid-state fermentation.  Table 2 presents the amount of acetic acid produced by different filamentous fungi (*A. terreus*). Acetic acid produced from *A. terreus* strains, which were isolated from locations of different agroecologic zones of north Gondar, Amhara regional state, Ethiopia, was recorded for 7 days. Acetic acid production rate varied among the fungal isolates at different incubation time periods.

A maximum mean value of 72.5 ± 1.65 g/l acetic acid was produced from an isolate from low-land agricultural soil (isolate LL_2_) in the north Gondar zone after 7 days of incubation. A mean value of 69.27 ± 0.00 g/l and 63.22 ± 0.64 g/l acetic acid was recorded from *A. terreus* isolates from midland (isolate ML_1_) to high-land (isolate HL_4_) agricultural soils of north Gondar zone, respectively. *A. terreus* isolate LL_2_ was chosen for further optimization studies as it produced the highest mean value of acetic acid from all the isolates.

### 3.4. Acetic Acid Generation and pH

**Figure 3:**
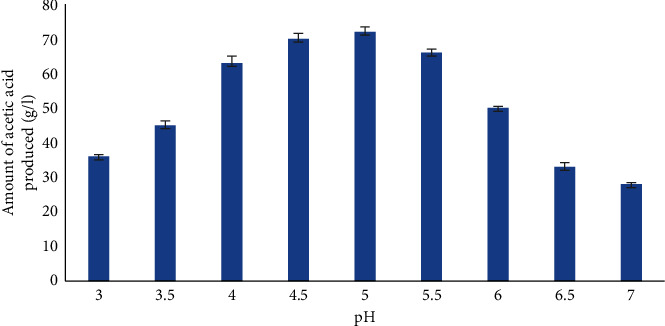
Acetic acid production by *A. terreus* isolate LL_2_ at different pH values. The values are expressed as mean ± standard deviation.

Solid-state fermentation is impacted by the pH of the medium. As demonstrated in Figure 3, an initial pH of 5 in this investigation was ideal for the formation of acetic acid. After fermentation, maximum acetic acid production (72.5 ± 1.11 g/l) was obtained by the selected isolate. There was a significant increase in acetic acid production as pH was decreased to 4.5 but further increase to pH 7 showed decreased acetic acid yield.

### 3.5. Effect of Ethanol Concentration on Acetic Acid Production

**Figure 4:**
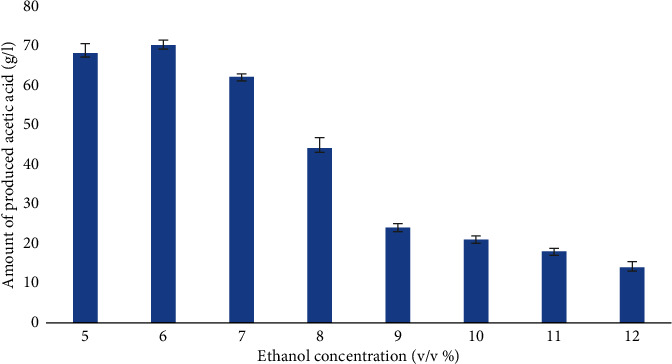
Acetic acid production by *A. terreus* isolate LL_2_ at different ethanol concentrations (%). The values are expressed as mean ± standard deviation.

Ethanol concentration affects the performance of fungi in the fermentation medium. In this study, the production of acetic acid at different ethanol concentrations is depicted in Figure 4. The yield of acetic acid revealed an obvious decrease with increasing ethanol supplementation (from 6 to 12%) after 6 days and reached up to a maximum amount of 70 ± 1.75 g/l, and then exhibited a gradual decrease.

### 3.6. Effect of Growth Temperature on the Production of Acetic Acid

**Figure 5:**
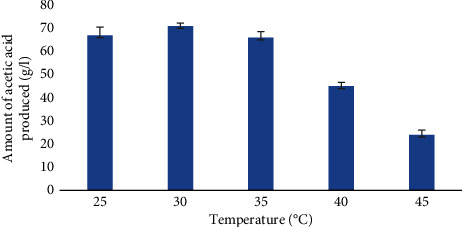
Acetic acid yield of *A. terreus* isolate from isolate LL_2_ at various temperatures. The values are expressed as mean ± standard deviation.

Figure 5 depicts the impact of temperature on the synthesis of acetic acid. The influence of temperature on acetic acid production was studied by carrying fermentation at temperature ranging from 25 to 45°C with five intervals. As per the result, maximum acetic acid production yield of 71 ± 1.3 g/l was obtained at 30°C. At a temperature of 45°C, lower concentration of acetic acid (24 ± 2 g/l) was produced. Thus, the optimum temperature for acetic acid production in this study was 30°C.

### 3.7. Sequence Similarity

**Figure 6:**
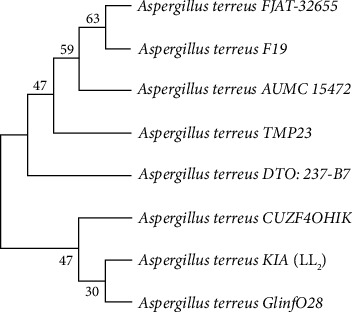
Phylogenetic tree of the ITS1-5.8 S-ITS2 rDNA region gene sequence of fungal isolate LL_2_ with related *A. terreus* species in NCBI database. The neighbor-joining (NJ) method of MEGA 11 software was used to construct the tree, and the evolutionary distances were computed with maximum composite likelihood method. The branching patterns were checked using 1000-bootstrap replicates. LL_2_ was given accession number of OR038984. The accession number of LL2 isolate is given in the name of KIA.

The fungal isolate obtained from low-land agricultural soil of north Gondar zone, Ethiopia (isolate LL_2_), was identified using different cultural and morphological characteristics. This isolate that produced appreciable amount of acetic acid was further confirmed by molecular characterization through the amplification and sequencing of the intergenic ITS (ITS1 and ITS2) and intervening 5.8S rDNA regions. *A. terreus* ITS1-5.8 S-ITS2 rDNA region was amplified and run on a 1.5% agarose gel to detect the isolates with the expected band size. The obtained DNA bands from the fungal isolates were observed to have a size of 500 bp. The sequence similarity of the ITS and 5.8S rDNA regions of isolate LL_2_ (KIA) to some of the *A. terreus* sequences in the NCBI database is shown in Figure 6. Isolate LL_2_ or KIA (Acc. No: OR038984), which produced the maximum amount of acetic acid, was amplified, sequenced, and compared to a strain sequence that retrieved from the GenBank database and found to exhibit 98.5% similarity with other *A. terreus* species (Gjinfo28).

## 4. Discussion

Organic acids have been used for many years in the food, chemical, agriculture, and pharmaceutical industries. These organic acids can be used as basic compounds for a wide variety of chemical industries such as in the production of polymers and various solvents. They differ on the basis of the involvement of carbon, hydrogen, and oxygen elements. Major types of organic acids produced by microbial activity, which represent a rising chemical segment, in which several bio-based compounds synthesized from them are citric, succinic, lactic, itaconic, lactobionic, gluconic, fumaric, propionic, and acetic acids [19].

This study aimed to identify acetic acid-producing fungi in several naturally occurring agricultural soil sources. Therefore, the researchers focused on several decomposed soil samples to look for potential acetic acid producing fungi. From a total of 50 soil samples, about 15 colonies were obtained on PDA agar plates.

Concerning morphological features of *A. terreus*, early growth of the isolates on PDA exhibited white mycelia, which gradually turned to velvety thick yellow soluble pigment-colored spores upon maturation, and the conidial head showed brown to buff to cinnamon-colored colonies. Microscopic examination revealed distinct conidiophore, which ended with a swollen vesicle bearing smooth-walled, globose-shaped as shown in Figure 1.

Strains were primarily screened and detected as acetic acid producing fungi because they produced yellow color around their tubs in the selective Czapek-Dox broth culture medium. The isolates were further checked for their abilities to produce acetic acid by titration. There are several *Aspergillus* and *Penicillium* species in nature, some of which produce organic acids. Numerous workers in various mine regions across the world have reported the finding of *A. niger*, *A. terreus*, *A. flavus*, *P. brevicompactum*, *P. oxalicum*, *P. purpurescens*, *P. lividum*, *Eupenicillium ludwigii*, and *P. spinulosum* [18, 20, 21].

Acetic acid yields between 72.5 and 29 g/l were obtained from *A. terreus* isolated from low-land agricultural soil in the north Gondar zone of Ethiopia. For cultures of 72.5 g/l acetic acid, the maximum productivity period lasted for 6 days. Because of its ability to metabolize a wide range of substrates and its demonstrated durability as a microorganism, *A. terreus* is the best option for use in processes using bio-based substrates. By examining the growth patterns and a number of features related to the synthesis of acetic acid by this isolate using substrates like barley straw, it is possible to produce this organic acid in bulk. Similar work was reported by Romero et al. [22].

The maximal acetic acid yield by *A. terreus* isolates from low-land (LL_2_), midland (ML_1_), and high-land (HL_4_) agricultural soils of north Gondar zone were 72.5 ± 1.65 g/l, 69.27 ± 0.00 g/l, and 63.22 ± 0.64 g/l, respectively. In fact, this result showed that the isolate from low-land agricultural soil had the highest mean value of acetic acid production capacity. This figure was somewhat better than the one reported by Miranda et al. [23] who reported 50.6 g/l acetic acid. The other research report was by Freer et al. [24] saying that a medium containing either 100 g/l of glucose or 35 g/l of ethanol as a carbon/energy source for a yeast strain *Dekkera intermedia* NRRL-4553 produced 42.8 and 14.9 g/l acetic acid from the two carbon sources, respectively, after 64.5 hours. The optimal pH was determined to be 5. When the initial glucose concentration was 150 or 200 g, the yeast produced 57.5 and 65.1 g/l acetic acid, respectively. Bovonsombut et al. [25] also reported an acetic acid synthesis of 4.06 g/l from acetic acid bacteria isolated from fruits.

For the optimization of acetic acid production, different ethanol concentrations (6, 7, 8, 9, 10, 11, and 12%) were tested. With these conditions, concentrations of up to 6–12% are possible. As ethanol concentrations increase from 6 to 10%, we impede culture development, which decreased productivity.

The growth of microorganisms is directly influenced by the pH of the culture medium and the chemical processes that the microorganisms perform. In the current study, acetic acid generation increases along with an increase in pH. At pH 5, where maximum production was observed, the best results were obtained.

The cultures were also held at 25, 30, 35, 40, and 45°C temperatures with 6% initial ethanol concentration, and fermentation was carried out for 6 days to determine the best temperature for acetic acid production. At 30°C, the yield was at its highest. However, the acid content reduced as the temperature was raised to 40°C and beyond. The decrease in acetic acid production with further increase in temperature was probably due to low enzyme activity. Thus, it was decided that 30°C was the best temperature for acetic acid production. This result is in agreement with the result reported by Egbe et al. [10].

The results in the current research work suggest that much more organic acid (especially acetic acid) producing filamentous fungi can be isolated from north Gondar zone agricultural soil. The potential strain (LL_2_/KIA) that was isolated in this study has good potential to be used for the production of acetic acid at the industrial level. Conventional identification method (morphological) must be complemented with molecular of identification, especially the use of ITS1-5·8S-ITS2 region gene sequencing. The ITS1-5.8S-ITS2 sequence of isolate LL_2_ was compared against the ones retrieved from the NCBI database. LL_2_ (Acc. No: OR038984) from low-land agricultural soil in north Gondar zone of Ethiopia in this study was found to be 98.5% similar to an *A. terreus* isolate in the GenBank. Sequence analysis of ITS 1-5.8S-ITS 2 was applied in identifying *A. niger* KW for the production of citric acid from agricultural wastes [8]. Accensi et al. [26] were also able to identify *A. niger* aggregate (*A. niger and A. tubingensis*) using ITS 1-5.8s-ITS 2 gene sequencing.

The current study showed that barely straw was found to be best substrate for the production of acetic acid from *A. terreus.* The use of natural substrates such as rice, split pea, and millet is consistent with research from Bapat et al. [27], in which *Aspergillus* species cultures were tested for citric acid generation. Of these cultures, *A. niger* S-6 generated the most citric acid, 0.33 g/100 ml. The use of barley straw that had been treated with 5 N HCl was shown to enhance the concentration of acetic acid. Our findings on the use of barley straw as a substrate for the production of acetic acid are consistent with the findings of previous researchers who examined the use of various other agricultural wastes as suitable and cost-effective substrates for the production of various organic acids such as acetic acid and citric acid by solid-state fermentation, such as cassava bagasse, apple pomace, coffee husk, pineapple waste, sugar beet cosset, African apple peel, and molasses [28–35].

At last, a table comparing the maximum yield in this study and previously published data is depicted below (Table 3).

## 5. Conclusion

This study demonstrates the possibility of isolating highly effective acetic acid producing fungus from soil. According to this study, using barley straw as a substrate for *A*. *terreus* low land (isolate LL_2_ or KIA) to produce acetic acid could be an effective way to reduce the challenges associated with disposing of barley straw while creating a less expensive, economically valuable organic acid. On the other hand, the optimum temperature and pH for the maximum production of acetic acid by *A*. *terreus* low land (isolate LL_2_ or KIA) were found to be 30°C and pH 5. The findings also revealed that some farms in low-land areas have soils that contain strains of *A*. *terreus* that can produce a fair amount of acetic acid. These fungal isolates could be used to produce acetic acid from inexpensive agricultural wastes and residues, such as wheat and barley straw.

## Figures and Tables

**Table 1:** Altitude, latitude, and longitude of sample collection sites.

Sample collection sites		Altitude (m)	Latitude and longitude
High-land	Debark	2.850	13°9′22″N, 37° 53′ 53″E
	Amba Giyorgis	2960.71	12°46′6.36″N, 37°37′31.28″E

Midland	Chilga	2146	12° 33′ 0″N, 37° 4′ 0″E
	Gondar	2133	12°35′59.99″N, 37°27′59.99″E
	Tikil Dingay	2243.65	12° 59′ 3″N, 37° 2′ 39″E
	Kola Diba	2146	12°25′21.70″N, 37°19′26.18″E

Low-land	Quara	1713	12° 18′0″N, 36°13′0″E
	Soroka	849	64°31′31″N, 34°45′56″E
	Metema	685	12°57′16.06″N, 36°9′26.12″E

**Table 2:** Mean acetic acid production (g/l) by *A. terreus* isolates from agricultural soils collected from different locations of north Gondar zone. The values are expressed as mean ± standard deviation.

Days	Acetic acid production (g/l)		
	Isolate HL_4_	Isolate LL_2_	Isolate ML1
1	20 ± 1.11^f^	29 ± 0.05^f^	26 ± 0.55^f^
2	34 ± 2.05^e^	43 ± 2.00^e^	40 ± 1.00^e^
3	49 ± 0.55^d^	58 ± 1.23^d^	55 ± 2.00^d^
4	57 ± 3.14^c^	66 ± 2.05^c^	63 ± 1.05^c^
5	61 ± 1.77^b^	70 ± 0.64^b^	67 ± 0.96^b^
6	63.22 ± 0.64^a^	72.5 ± 1.65^a^	69.27 ± 0.00^a^
7	60 ± 0.75^b^	66.5 ± 2.15^c^	65 ± 3.11^b^

Values are mean ± SD of 3 replicates. Values followed by different superscripts are significantly different (*P* ≤ 0.05). Values followed by the same superscripts are not significantly different (*P* ≤ 0.05). The purpose these letters is whether there is significance difference among treatment means or not.

**Table 3:** A comparative table comparing *A. terreus* isolate LL2 yield with previously published data.

Production of acetic acid by *A. terreus* isolate LL2	Previous work reports	References
72.5 ± 1.65 g/l	50.6 g/l	Miranda et al. [23]
	42.8 and 14.9 g/l	Freer et al. [24]
	4.06 g/l	Bovonsombut et al. [25]
	7.1 ± 0.2 g/l	Lei [36]
	7.0 g/100 ml	Islam et al. [37]

## Data Availability

The data used to support the findings of this study are included in the article.
